# Evaluation of the efficiency of genomic versus pedigree predictions for growth and wood quality traits in Scots pine

**DOI:** 10.1186/s12864-020-07188-4

**Published:** 2020-11-16

**Authors:** Ainhoa Calleja-Rodriguez, Jin Pan, Tomas Funda, Zhiqiang Chen, John Baison, Fikret Isik, Sara Abrahamsson, Harry X. Wu

**Affiliations:** 1 Skogforsk (The Forestry Research Institute of Sweden), Box 3, Sävar, SE 918 21 Sweden; 2 Umeå Plant Science Centre, Department of Forest Genetics and Plant Physiology, Swedish University of Agricultural Sciences, Umeå, SE - 901 83 Sweden; 3 Department of Genetics and Breeding, Faculty of Agrobiology and Natural Resources, Czech University of Life Sciences Prague, Prague, 165 00 Czech Republic; 4 Key Laboratory of Forest Genetics and Biotechnology, Nanjing Forestry University, Nanjing, 210037 China; 5 RAGT Seeds, Essex, CB 101TA United Kingdom; 6 Department of Forestry and Environmental Resources, North Carolina State University, Raleigh, NC 27695 USA; 7 Beijing Advanced Innovation Centre for Tree Breeding by Molecular Design, Beijing Forestry University, Beijing, 100083 China; 8 National Research Collection Australia, CSIRO, Canberra, ACT 2601 Australia

**Keywords:** *Pinus sylvestris*, genotyping-by-sequencing, GBLUP, Bayesian, predictive ability, predictive accuracy, theoretical accuracy, prediction accuracy

## Abstract

**Background:**

Genomic selection (GS) or genomic prediction is a promising approach for tree breeding to obtain higher genetic gains by shortening time of progeny testing in breeding programs. As proof-of-concept for Scots pine (*Pinus sylvestris* L.), a genomic prediction study was conducted with 694 individuals representing 183 full-sib families that were genotyped with genotyping-by-sequencing (GBS) and phenotyped for growth and wood quality traits. 8719 SNPs were used to compare different genomic with pedigree prediction models. Additionally, four prediction efficiency methods were used to evaluate the impact of genomic breeding value estimations by assigning diverse ratios of training and validation sets, as well as several subsets of SNP markers.

**Results:**

Genomic Best Linear Unbiased Prediction (GBLUP) and Bayesian Ridge Regression (BRR) combined with expectation maximization (EM) imputation algorithm showed slightly higher prediction efficiencies than Pedigree Best Linear Unbiased Prediction (PBLUP) and Bayesian LASSO, with some exceptions. A subset of approximately 6000 SNP markers, was enough to provide similar prediction efficiencies as the full set of 8719 markers. Additionally, prediction efficiencies of genomic models were enough to achieve a higher selection response, that varied between 50-143% higher than the traditional pedigree-based selection.

**Conclusions:**

Although prediction efficiencies were similar for genomic and pedigree models, the relative selection response was doubled for genomic models by assuming that earlier selections can be done at the seedling stage, reducing the progeny testing time, thus shortening the breeding cycle length roughly by 50%.

**Supplementary Information:**

The online version contains supplementary material available at (doi:10.1186/s12864-020-07188-4).

## Background

Genomic prediction or genomic selection (GS) was proposed by Meuwissen et al. [1] as a methodology to use genome-wide dense marker information to estimate genetic values for selection of breeding populations. The main difference between GS and previous approaches, such as marker-assisted selection (MAS), is that in MAS a requirement is to identify quantitative trait loci (QTL) first by linkage disequilibrium (LD) in breeding varieties and then use them as candidate genes for selection, whereas in GS it is not necessary to detect QTL and their significance, using markers prior to selection [2].

The application of GS requires phenotypic data and marker information of a training population (TP) of individuals that are used to develop prediction models to calculate genomic estimated breeding values (GEBV). GEBV are then validated through a validation population (VP) of individuals, or selection candidates, which are genetically related to the TP, and for which only marker data is available to predict their own GEBV [3–5]. Since the introduction of GS, many simulations and experimental results in animal-, crop- and tree breeding have shown the potential of GS to estimate genetic values, to shorten breeding cycles, to increase selection intensities and capture Mendelian segregation effects in order to increase genetic gains [6–8].

Practical application of GS in animal and crop breeding programs would not have been possible without the rapid and cost-effective development of next-generation sequencing (NGS) technologies which, consequently, have accelerated the discovery of thousands of single nucleotide polymorphisms (SNP) markers [9–11]. Among all the NGS technologies nowadays available, genome-wide SNP arrays had been shown as preferable for their reproducibility, manageability and storage logistics, as well as their cost efficiency for breeding programs [2]. However, genome-wide SNP arrays require the availability of a reference genome to compare, contrast and detect SNP markers from the genome of the population of interest.

The size and complexity of the conifer mega-genomes (20–40 Gbp), makes the genome assembly process tedious and costly; so far only a few conifer genomes have been assembled, for instance, *Picea glauca* (Moench) Voss [12], *Picea abies* (L.) Karst [13], *Pinus taeda* L. [14, 15], *Pinus lambertiana* Dougl. [16], and recently, the first draft of the *Pinus radiata* D. Don genome (G. Sturrock personal communication). Hence, SNP arrays from genome re-sequencing are not available for most conifer species and other technologies as genotyping-by-sequencing (GBS) and/or exome probe panels have been used instead [17–22].

Scots pine (*Pinus sylvestris* L.) is the most widely distributed pine in the world [23, 24]. It is also a highly important commercial species in Europe, particularly in northern countries [25], being the second foremost species for wood production in Sweden [26]. Despite its importance, neither a reference genome, nor a SNP array, are at present available for the species. While the first draft of the Scots pine genome is currently ongoing [27], and until a SNP-chip is developed for the species, it is necessary to use other NGS methodologies in the meantime.

GBS uses restriction enzymes based complexity reduction sequencing method suited for complex, large genomes. GBS utilizes a barcoding system for multiplex sequencing, which increases its efficiency and reduces the genotyping costs [28, 29]. GBS can generate a very large number of SNPs but also produces significant amount of missing data. The latter can be solved with the aid of different imputation methods, such as mean imputation (MI), expectation maximization (EM), family-based k-nearest neighbor (kNN-Fam) or singular value decomposition (SVD) [30, 31]. The imputation with EM algorithm developed at the R package rrBLUP, was specially designed for GBS data assuming that markers follow a multivariate normal distribution and are imputed based on a realized relationship (averaged over all markers), resulting in higher accuracies of GEBV [32, 33]. GBS marker information has been successfully used for parentage reconstruction in Scots pine [34], as well as to perform genomic predictions studies in livestock [35, 36], maize [37], wheat [33], soybean [38], *Picea glauca* (Moench) Voss ×*Picea engelmannii* Parry ex Engelm. [39] and radiata pine [40]. Therefore, GBS is an attractive technology that can be used to perform GS and genome-wide association studies (GWAS) for Scots pine [41, 42].

A typical Scots pine breeding program consists of a combination of several selection strategies, essentially conventional progeny testing and breeding value prediction based on pedigree information and reliable phenotypic assessments, at age of 10–15 years. Usually the breeding cycle takes roughly 36 years when the selection strategy is backward selection based on polycross progeny tests of full-sibs, or 21 years when the strategy is forward selection [43]. One of the greatest advantages of GS in conifers is the potential to reduce the length of the breeding cycle, for example by shortening field progeny test time through early evaluation of greenhouse seedlings, based on molecular marker information. Furthermore, selection intensities can increase and therefore higher genetic gains per unit time could be achieved [2, 44, 45].

Traditional breeding value predictions consist on generating kinship coefficients between relatives to estimate the numerator relationship matrix (NRM), based on pedigree, i.e., a relationship matrix based on the expected proportion of the genome shared by two individuals [46, 47]. The NRM is then used in a Best Linear Unbiased Prediction (BLUP) analysis [48] to calculate Estimated Breeding Values (EBV). On the other hand, EBV can be estimated by replacing the NRM in the BLUP analysis with a genomic realized relationship matrix (GRM or RRM), generated with the kinship coefficients or realized proportion of the genome shared between individuals, computed based on the marker information, i.e., the number of loci shared between individuals [45, 49]. Hence, relationships between individuals are more accurately estimated given that the markers can account for Mendelian inconsistencies and for the contemporary and historical pedigree [2, 50, 51], if the number of markers is enough to path the identical-by-descent (IBD) status across the genome [52].

Multiple statistical methods are available to estimate GEBV. Genomic Best Linear Unbiased Prediction (GBLUP) is based on coancestry and the infinitesimal model in quantitative genetics, assuming that QTL allelic effects are normally distributed, and all have a similar contribution to the genetic variance. Conversely, most of the Bayesian approaches presume a prior non-normal distribution of QTL allelic effects (gamma or exponential distribution), thus the variance at each locus can vary [1, 49, 53]. For instance, Bayesian LASSO (BL) assumes that QTL effects follow a Laplace (or double exponential) distribution [54]. Nevertheless, Bayesian ridge regression (BRR) assigns QTL effects to a multivariate normal prior distribution with a common variance, which is modelled hierarchically through a scaled inverted chi-squared distribution [53, 55, 56].

The accuracy of GS predictions depends on the model selected, but also on other factors such as the level of LD, heritability of the trait, effective population size (Ne), TP size, density and amount of the SNP markers, and distribution of QTL effects [3, 7, 57]. Generally large Ne (i.e., low LD between SNP markers and QTL) normally decreases the precision of the GS models, as well as a small number of individuals in the TP or the low heritability of the trait of interest [45, 58, 59]. Increasing the number of high density markers and the size of TP can improve the efficiency of the GS models to a certain extent [60–62].

Despite the great number of articles published during the recent years on genomic prediction on forest species, different methodologies have been used to assess the effectiveness or accuracy of predictions, which complicates the comparison of different models and reliabilities between them. By definition, accuracy is the correlation between true breeding values (TBV) and EBV [63], but TBV are never known, therefore approximations to TBV need to be used. The most common methods used in tree breeding to estimate efficiency of genomic prediction models are, 1) the predictive ability (*r*_1_) estimated as the Pearson product-moment correlation between the cross-validated GEBV and phenotypes, 2) predictive accuracy (*r*_2_) estimated as *r*_1_ scaled by the square root of heritability, 3) theoretical accuracy (*r*_3_) which is the square root of reliability (i.e., squared correlation between TBV and EBV) [63, 64], and 4) the Accuracy or prediction accuracy (*r*_4_) defined as the Pearson product-moment correlation between the cross-validated GEBV and the pedigree based EBV (PEBV) estimated from PBLUP (pedigree based Best Linear Unbiased Prediction). Generally, *r*_4_ showed the highest values whereas *r*_1_ showed the lowest ones, when compared with the remaining methods, for instance in eucalypt hybrids (*Eucalyptus urophylla* ×*Eucalyptus grandis*) [65], maritime pine (*Pinus pinaster* Ait.) [66, 67], Norway spruce [68], or *Eucalyptus nitens* [69].

The genomic model may influence the effectiveness of the estimates and Bayesian approaches may seem more appropriate as they can accommodate different distributions of the allelic effects, however the literature on GS in forest trees showed similar results for most models. For instance, Chen et al. [68] observed similar *r*_1_ and *r*_4_ among four genomic prediction models (GBLUP, BRR, BL and reproducing kernel hilbert space (RKHS) in Norway spruce. Isik et al. [66] detected similar *r*_1_ in maritime pine comparing GBLUP, BRR and BL prediction models. Although GBLUP and ridge regression BLUP (rrBLUP) were recommended by Tan et al. [61] for their computational advantages in a eucalypt hybrid study, similar *r*_1_ were noted for GBLUP, rrBLUP, BL and RKHS. In an interior spruce study, Ratcliffe et al. [70] stated similar *r*_4_ for rrBLUP and BayesC *π*, which in turn performed better than the generalized ridge regression (GRR), whereas Thistlethwaite et al. [71] observed almost identical predictions with rrBLUP and GRR in Douglas-fir (*Pseudotsuga menziensii* Mirb. (Franco)). On the contrary, Resende et al. [58] observed better *r*_1_ for disease resistance in a loblolly pine study with Bayesian methods when compared with BLUP-based methods.

The objective of this study was to assess, as proof-of-concept for Scots pine, the effectiveness (or efficiency) of genomic versus pedigree predictions for growth and wood quality traits, using two imputation algorithms combined with four prediction models (GBLUP, BL, BRR and PBLUP) and comparing four methods to assess efficiencies (*r*_1_,*r*_2_,*r*_3_ and *r*_4_) under several training and validation population scenarios as well as with different numbers of SNPs.

## Results

### Heritabilities

Narrow sense heritability estimates based on PBLUP were slightly higher than those based on GBLUP, excluding DBH2 (diameter at breast height assessed at 36 years old) which was higher for GBLUP-EM (Table 1). MOEs showed the same heritability for PBLUP and GBLUP-EM. GBLUP heritability estimates calculated from the RRM derived from EM imputation method were higher than those derived from the RND imputation method for almost all traits, except Ht1 (tree height measured at 10 years old) and MOEd. Standard errors were similar for growth traits regardless of the BLUP method used but they were always lower when derived from GBLUP methods.

**Table 1:** Additive genetic variance $\left (\widehat {\sigma }_{a}^{2}\right)$, residual variance $\left (\widehat {\sigma }_{e}^{2}\right)$ and narrow sense heritability with standard errors $\left (\widehat {h}^{2}\pm \text {SE}\right)$, from PBLUP and GBLUP models for eight phenotypic traits

Trait	Model	$\widehat {\sigma }_{a}^{2}$	$\widehat {\sigma }_{e}^{2}$	$\widehat {h}^{2}\pm $SE
Ht1	PBLUP	331.3	1445.9	0.19 ±0.06
	GBLUP-EM	294.6	1504.6	0.16 ±0.06
	GBLUP-RND	305.2	1484.3	0.17 ±0.06
Ht2	PBLUP	3827.5	5810.3	0.40 ±0.09
	GBLUP-EM	3539.0	6170.3	0.37 ±0.08
	GBLUP-RND	3437.0	6075.4	0.36 ±0.08
DBH1	PBLUP	147.2	460.6	0.24 ±0.07
	GBLUP-EM	144.7	473.4	0.23 ±0.07
	GBLUP-RND	133.6	475.4	0.22 ±0.07
DBH2	PBLUP	158.8	628.7	0.20 ±0.07
	GBLUP-EM	173.4	625.6	0.22 ±0.07
	GBLUP-RND	164.4	624.2	0.21 ±0.06
MFA	PBLUP	4.8	12.4	0.28 ±0.08
	GBLUP-EM	4.3	13.3	0.24 ±0.07
	GBLUP-RND	4.0	13.3	0.23 ±0.07
MOEs	PBLUP	1.3	2.0	0.39 ±0.10
	GBLUP-EM	1.4	2.1	0.39 ±0.09
	GBLUP-RND	1.2	2.2	0.35 ±0.08
DEN	PBLUP	419.0	543.9	0.44 ±0.10
	GBLUP-RM	402.9	593.3	0.40 ±0.09
	GBLUP-RND	367.7	595.6	0.38 ±0.08
MOEd	PBLUP	0.8	1.0	0.46 ±0.10
	GBLUP-EM	0.7	1.1	0.38 ±0.08
	GBLUP-RND	0.7	1.1	0.39 ±0.08

**Fig. 1:**
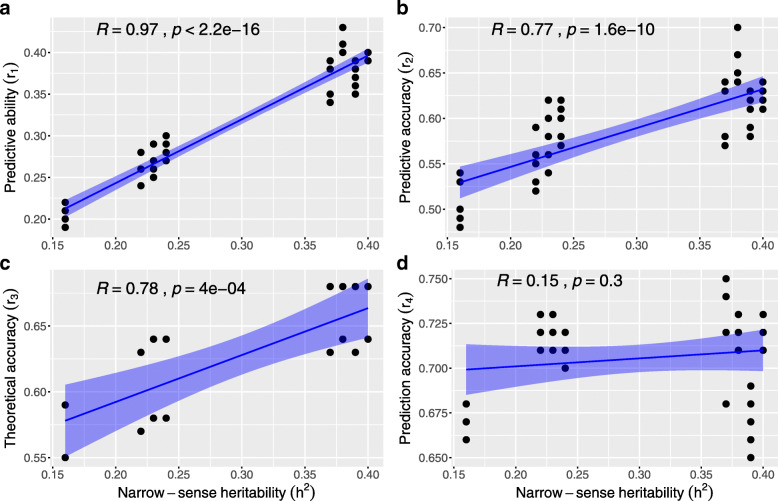
Regression plots between all genomic models prediction efficiencies and narrow sense heritability ($\widehat {h}^{2}$). Prediction efficiencies: **a**) *r*_1_ - predictive ability, **b**) *r*_2_ - predictive accuracy, **c**) *r*_3_ - theoretical accuracy, and **d**) *r*_4_ - prediction accuracy

Among the genomic models, *r*_1_,*r*_2_ and *r*_3_ were larger for traits with higher narrow sense heritabilities (Ht2, DEN, MOEd and MOEs) than for traits with low narrow sense heritabilities (Ht1, DBH1, DBH2 or MFA microfibril angle) (Table 2). Moreover, across all genomic models and imputation methods, a positive linear correlation between *r*_1_,*r*_2_,*r*_3_ and trait heritabilities was observed (respectively r=0.97, p <0.0001; r=0.77, p <0.0001 and r=0.78, p <0.0001), but not between *r*_4_ and heritabilities (r=0.15, p=0.3) (Fig. 1).

**Table 2:** Prediction efficiencies of genetic models for eight phenotypic traits. Four prediction efficiencies (*r*_1_ - predictive ability, *r*_2_ - predictive accuracy, *r*_3_ - theoretical accuracy, and *r*_4_ - prediction accuracy, and their standard errors) for eight traits based on pedigree (PBLUP), and three genomic models (GBLUP, BL, and BRR) combined with two imputation methods (EM and RND)

		Traits							
**Model**	Pred. eff.	Ht1	Ht2	DBH1	DBH2	MFA	MOEs	DEN	MOEd
PBLUP	*r*_1_	0.21 ±0.00	0.37 ±0.00	0.27 ±0.00	0.23 ±0.04	0.31 ±0.00	0.39 ±0.00	0.41 ±0.00	0.44 ±0.00
	*r*_2_	0.54 ±0.01	0.61 ±0.01	0.55 ±0.01	0.49 ±0.01	0.63 ±0.01	0.62 ±0.00	0.65 ±0.00	0.71 ±0.00
	*r*_3_	0.52 ±0.00	0.60 ±0.00	0.55 ±0.00	0.53 ±0.00	0.57 ±0.00	0.60 ±0.00	0.61 ±0.00	0.62 ±0.00
	*r*_4_	0.84 ±0.00	0.80 ±0.00	0.84 ±0.00	0.84 ±0.00	0.84 ±0.00	0.75 ±0.00	0.81 ±0.00	0.82 ±0.00
GBLUP-EM	*r*_1_	0.20 ±0.00	0.39 ±0.00	0.26 ±0.00	0.26 ±0.00	0.29 ±0.00	0.38 ±0.00	0.40 ±0.00	0.41 ±0.00
	*r*_2_	0.49 ±0.00	0.64 ±0.01	0.56 ±0.01	0.55 ±0.01	0.60 ±0.01	0.61 ±0.00	0.63 ±0.00	0.67 ±0.00
	*r*_3_	0.59 ±0.00	0.68 ±0.00	0.64 ±0.00	0.63 ±0.00	0.64 ±0.00	0.68 ±0.00	0.68 ±0.00	0.68 ±0.00
	*r*_4_	0.68 ±0.00	0.75 ±0.00	0.73 ±0.00	0.73 ±0.00	0.72 ±0.00	0.69 ±0.00	0.73 ±0.00	0.73 ±0.00
GBLUP-RND	*r*_1_	0.19 ±0.00	0.38 ±0.00	0.26 ±0.00	0.24 ±0.00	0.28 ±0.00	0.37 ±0.00	0.39 ±0.00	0.40 ±0.00
	*r*_2_	0.48 ±0.01	0.63 ±0.01	0.54 ±0.01	0.52 ±0.01	0.57 ±0.01	0.59 ±0.00	0.61 ±0.00	0.65 ±0.00
	*r*_3_	0.55 ±0.00	0.63 ±0.00	0.58 ±0.00	0.57 ±0.00	0.58 ±0.00	0.63 ±0.00	0.64 ±0.00	0.64 ±0.00
	*r*_4_	0.66 ±0.00	0.74 ±0.00	0.72 ±0.00	0.71 ±0.00	0.71 ±0.00	0.67 ±0.00	0.71 ±0.00	0.71 ±0.00
BL-EM	*r*_1_	0.20 ±0.04	0.34 ±0.01	0.29 ±0.04	0.26 ±0.03	0.28 ±0.03	0.36 ±0.04	0.39 ±0.03	0.40 ±0.03
	*r*_2_	0.50 ±0.10	0.57 ±0.02	0.60 ±0.08	0.56 ±0.07	0.58 ±0.06	0.58 ±0.06	0.61 ±0.05	0.65 ±0.05
	*r*_4_	0.67 ±0.03	0.68 ±0.01	0.73 ±0.02	0.72 ±0.02	0.70 ±0.02	0.66 ±0.02	0.72 ±0.02	0.71 ±0.02
BL-RND	*r*_1_	0.20 ±0.04	0.34 ±0.03	0.27 ±0.03	0.26 ±0.04	0.29 ±0.04	0.37 ±0.03	0.39 ±0.03	0.40 ±0.03
	*r*_2_	0.48 ±0.10	0.57 ±0.05	0.58 ±0.07	0.56 ±0.08	0.61 ±0.08	0.62 ±0.05	0.62 ±0.05	0.64 ±0.05
	*r*_4_	0.66 ±0.02	0.72 ±0.02	0.73 ±0.02	0.72 ±0.02	0.70 ±0.02	0.66 ±0.02	0.72 ±0.02	0.72 ±0.02
BRR-EM	*r*_1_	0.21 ±0.03	0.35 ±0.01	0.29 ±0.03	0.28 ±0.03	0.30 ±0.03	0.39 ±0.03	0.40 ±0.03	0.43 ±0.03
	*r*_2_	0.53 ±0.09	0.58 ±0.02	0.62 ±0.07	0.59 ±0.07	0.62 ±0.07	0.63 ±0.05	0.62 ±0.05	0.70 ±0.05
	*r*_4_	0.67 ±0.02	0.68 ±0.01	0.73 ±0.02	0.72 ±0.02	0.72 ±0.02	0.68 ±0.02	0.72 ±0.02	0.73 ±0.02
BRR-RND	*r*_1_	0.22 ±0.04	0.39 ±0.03	0.25 ±0.03	0.24 ±0.04	0.27 ±0.04	0.35 ±0.03	0.39 ±0.03	0.40 ±0.03
	*r*_2_	0.54 ±0.10	0.64 ±0.05	0.54 ±0.07	0.53 ±0.09	0.57 ±0.08	0.59 ±0.05	0.63 ±0.05	0.64 ±0.04
	*r*_4_	0.67 ±0.02	0.75 ±0.02	0.71 ±0.02	0.71 ±0.02	0.70 ±0.02	0.65 ±0.02	0.71 ±0.02	0.71 ±0.02

### Prediction efficiency of the different models

Through 10-fold cross-validation, *r*_1_,*r*_2_ and *r*_4_ were estimated for all models and imputation methods, and additionally *r*_3_ was also estimated for GBLUP and PBLUP (Table 2). The *h* (square root of heritability) estimated from the GBLUP-EM using the full data, was used to calculate *r*_2_, since the RRM captures IBD and identical-by-state (IBS) status between the individuals and can be considered a better estimation than the one from PBLUP.

The lowest prediction efficiency estimates were obtained for *r*_1_ (0.19–0.44) and the highest for *r*_4_ (0.66–0.84) for all traits, regardless of the model and imputation method used (Table 2). The genomic prediction models performed similarly for all the different calculations of the efficiency (*r*_1_ to *r*_4_) for most traits, except in terms of *r*_2_ for which BRR-EM showed slightly higher estimations for wood traits and diameter, compared with the other genomic prediction methods. Concerning *r*_3_, GBLUP-EM showed higher estimations than GBLUP-RND and PBLUP for all traits. Similarly, among all the genomic models *r*_4_ showed higher values for GBLUP-EM for all traits. In summary, although the best *r*_4_ were observed with PBLUP for all traits, genomic prediction models performed similarly or slightly better than PBLUP regarding *r*_1_,*r*_2_ and *r*_3_ for all traits. Moreover, there was no single genomic prediction model that performed better than others for all the traits, and only EM imputation method combined with GBLUP or BRR had some improvement for some traits.

### Relative size effect of the training and validation populations

**Fig. 2:**
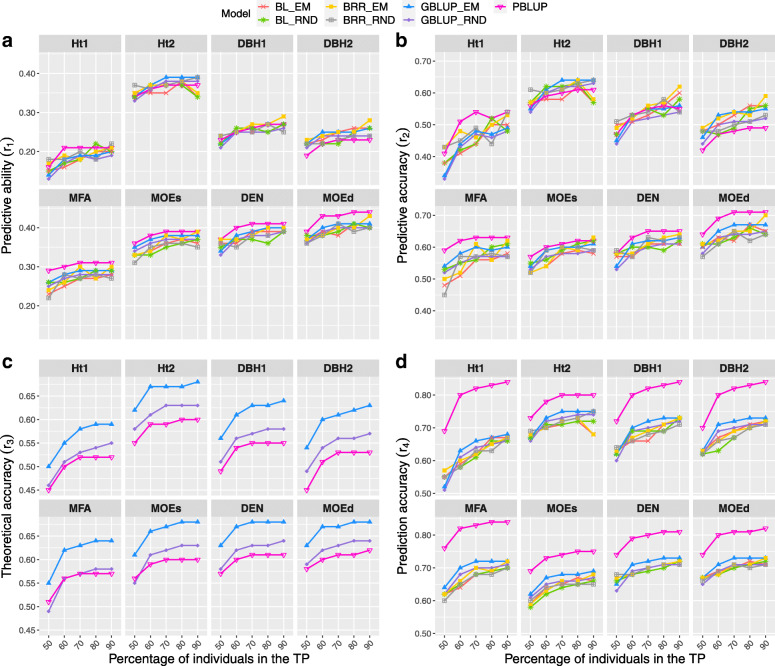
Prediction efficiencies of different training (TP) and validation population (VP) sizes. Prediction efficiencies: *r*_1_ - predictive ability, *r*_2_ - predictive accuracy, *r*_3_ - theoretical accuracy, and *r*_4_ - prediction accuracy. TP sizes: 50%, 60%, 70%, 80% and 90% of the total number of individuals

All models showed similar increasing patterns of *r*_1_,*r*_2_,*r*_3_ and *r*_4_ as the number of individuals in the TP increased, for all traits (Fig. 2). The lowest *r*_1_,*r*_2_,*r*_3_ and *r*_4_ were observed when half of the individuals were assigned to the TP. However, BRR-EM model only reached its highest *r*_1_,*r*_2_ and *r*_4_ when 90% of individuals were assigned at the TP, for all traits except Ht2 (height measured at 30 years old). In terms of *r*_1_ all the remaining models perform similarly when TP size was between 70–90% of individuals, for almost all traits (Fig. 2a).

No clear pattern was observed regarding *r*_2_, with the peak for genomic models when 90% of individuals were allocated to the TP, whereas PBLUP for wood traits, by contrast, showed a plateau for a TP containing 70–90% of the individuals (Fig. 2b). Concerning *r*_3_, similar efficiencies were seen when TP included 70 to 90% of individuals for GBLUP-EM, performing better than GBLUP-RND and PBLUP (Fig. 2c).

Among all methods, PBLUP had the highest *r*_4_ for all eight traits regardless of the TP ratio (Fig. 2d), whereas genomic models performed similarly. Nevertheless, unlike the Bayesian models and GBLUP-RND that required 80–90% of individuals to be allocated to the TP to reach the highest *r*_4_, GBLUP-EM needed a subsample of 70% or 80% individuals as TP for almost all traits.

### Effect of increasing number of markers on predictions

The impact of the different subsets of SNPs was tested on BRR-EM and BL-EM models since such models consider different distribution of the QTL allelic effects. For instance, the variance at each locus can change, thus the effect of the SNP subsets on the model and its ability to predict BVs can be easily observed. The EM imputation method was selected due to the slightly higher values showed in the previous sections.

**Fig. 3:**
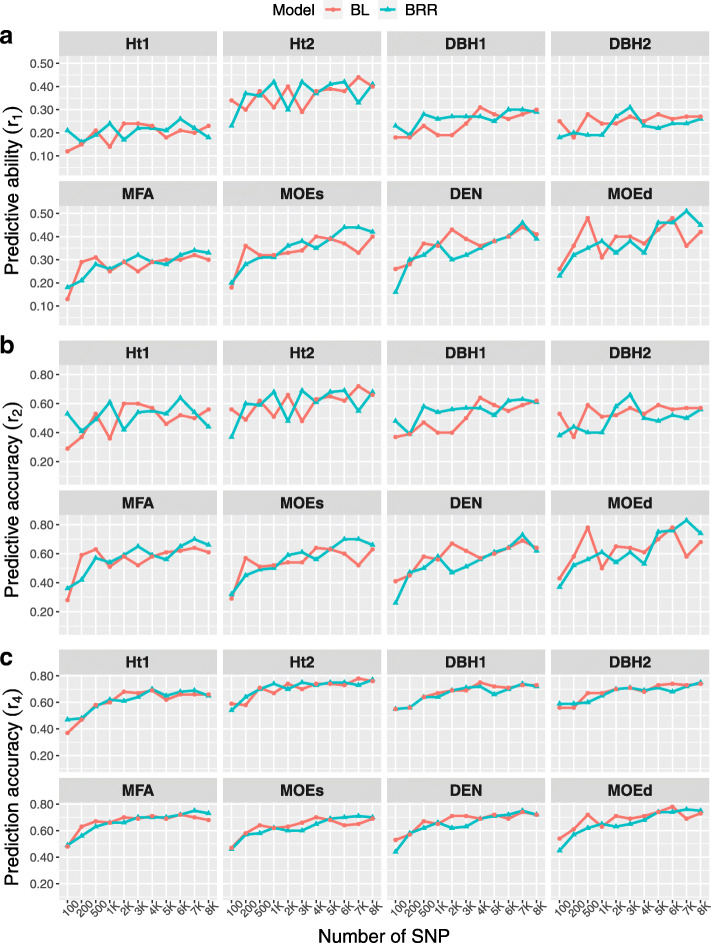
Prediction efficiencies of the number of markers. Prediction efficiencies: *r*_1_ - predictive ability, *r*_2_ - predictive accuracy, *r*_3_ - theoretical accuracy, and *r*_4_ - prediction accuracy. Eleven subsets of SNP markers (100, 200, 500, 1000, 2000, 3000, 4000, 5000, 6000, 7000 and 8719)

The *r*_1_,*r*_2_ and *r*_4_ increased for all traits as the number of SNPs rose (Fig. 3). However, for almost all traits, the greatest increase on *r*_1_ and *r*_2_ was attained when the subset of markers was 1000 SNPs, yet some oscillations were observed for both models at SNPs subsets of 500 to 6K for almost all traits (Fig. 3a and b). Although BRR and BL models had different patterns for the different number of SNPs, they showed similar *r*_1_ and *r*_2_ between 6K and 8K SNPs for most of the traits. Ht2, MOEs (static modulus of elasticity), DEN (density) and MOEd (dynamic modulus of elasticity) had the highest *r*_1_ and *r*_2_.

Conversely, *r*_4_ nearly followed an identical pattern of ascent for both models, as the number of SNPs increased (Fig. 3c). However, *r*_4_ kept almost constant around the same value at a range of 3K to 8k SNPs, for both models and all traits.

In short, for both models, the highest values of *r*_4_ and *r*_2_ were observed at SNP subsets that varied between 6K 8K, while within the range 3K 8K SNPs, no substantial increase was identified in *r*_4_ for any of the traits.

### Relative selection response of GS

The relative genomic selection response, *RSR*_*GS*:*PS*_, was estimated for each genomic selection model (GBLUP, BRR and BL) considering only the EM imputation method since this method showed equal or slightly higher efficiencies (between 0.00–0.07) than RND method. The Swedish Scots pine breeding cycle combines several selection strategies sorted in two groups, according to their cycle lengths [43]. For the first group of strategies (i.e., backward selection) the cycle length takes up to 36 years, whereas for the second group of strategies (i.e., forward selection) it takes up to 21 years. In order to estimate *RSR*_*GS*:*PS*_, it was assumed that GS could help to reduce the cycle lengths to 18 and 11 years following two approaches. The first approach assumed that the cycle could be reduced to 18 years, shortening the progeny test time but considering that female flowering starts at 15–18 years in Scots pine [24]. The second approach presupposed that earlier flowering greenhouse stimulation [72] would produce female flowering at an earlier age in Scots pine, thus the breeding cycle could be reduced to about 11 years. In addition and for both approaches a reduction in the progeny test time was also assumed.

The percentage of increase in selection efficiency for all traits and models showed the potential of GS when reducing the breeding cycle by 50% or more (Table 3). For backward selection strategy and aided by GS, a reduction of 50% in the breeding cycle length (i.e., from 36 to 18 years) resulted in percentages of increased selection efficiencies between 57.1%–143.5%. Moreover, a further reduction in the breeding cycle length of more than 50% (i.e., from 36 to 11 years) assisted by GS and flowering greenhouse stimulation, increased considerably the selection efficiency, being greater for prediction efficiencies *r*_1_,*r*_2_ and *r*_3_ (195.6%–298.4%) than for *r*_4_ (157.1%–206.8%). On the other hand, for forward selection, a reduction of 3 years in the breeding cycle aided by GS showed small percentages of increase in selection efficiencies for *r*_1_,*r*_2_ and *r*_2_ ((5.4%–42.0%). Furthermore, this small reduction in the cycle length, showed that traditional phenotypic selection would be more effective, for some traits, in terms of *r*_4_ given the low percentages of selection efficiencies showed (-8.3%–9.4%). Nevertheless, for forward selection strategy a reduction of 50% in the breeding cycle length (i.e. from 21 to 11 years) assisted by GS and flowering stimulation, increased the selection efficiencies for all prediction efficiency methods varying between 50%–126.9%. In summary, for all traits and genomic prediction models, the percentage of increase in selection efficiency exceeded 50% when the breeding cycle was reduced by 50%, reaching in many cases percentages that varied between 50%–143.5%.

**Table 3:** Percentage of increase in selection efficiency of GS for each phenotypic trait, estimated for both selection strategies (Strategy 1 and 2), model ratio (i.e., GBLUP/PBLUP, BRR/PBLUP and BL/PBLUP) and prediction efficiency (*r*_1_ - predictive ability, *r*_2_ - predictive accuracy, *r*_3_ - theoretical accuracy, and *r*_4_ - prediction accuracy). Approach 1 and 2 are respectively the breeding cycle length assumptions without and with flowering greenhouse stimulation (i.e. 18 and 11 years)

			Traits							
Selection	Ratio	Pred. eff.	Ht1	Ht2	DBH1	DBH2	MFA	MOEs	DEN	MOEd
Strategy 1	GBLUP/PBLUP	*r*_1_	90.5	110.8	92.6	126.1	87.1	94.9	95.2	86.4
(Approach 1)		*r*_2_	81.5	109.8	103.6	124.5	87.8	96.8	93.9	88.7
		*r*_3_	126.9	126.7	132.7	137.7	124.6	126.7	123.0	119.4
		*r*_4_	61.9	87.5	73.8	73.8	71.4	84.0	80.3	78.1
	BRR/PBLUP	*r*_1_	100.0	89.2	114.8	143.5	93.6	100.0	95.1	95.5
		*r*_2_	85.2	86.9	118.2	128.6	136.7	87.1	87.7	83.1
		*r*_4_	59.5	70.0	73.8	71.4	71.4	81.3	77.8	78.5
	BL/PBLUP	*r*_1_	90.5	83.8	114.8	126.1	80.7	84.6	90.2	81.8
		*r*_2_	100.0	109.8	96.4	116.3	132.7	90.3	93.9	80.3
		*r*_4_	57.1	80.0	73.8	71.4	66.7	76.0	77.8	75.6
Strategy 1	GBLUP/PBLUP	*r*_1_	211.7	245.0	215.2	270.0	206.2	219.9	219.3	205.0
(Approach 2)		*r*_2_	197.0	243.4	233.2	267.4	207.2	222.0	217.2	208.8
		*r*_3_	271.3	270.3	280.8	289.0	267.5	270.9	264.8	258.9
		*r*_4_	164.9	206.8	184.4	184.4	180.5	201.1	195.0	191.4
	BRR/PBLUP	*r*_1_	227.3	209.6	251.5	298.4	216.7	227.3	219.3	219.8
		*r*_2_	203.0	205.8	257.0	274.0	287.4	206.2	207.1	199.6
		*r*_4_	161.0	178.2	184.4	180.5	180.5	196.7	190.9	191.4
	BL/PBLUP	*r*_1_	211.7	200.7	251.5	270.0	195.6	202.1	211.3	197.5
		*r*_2_	227.3	243.4	221.3	254.0	280.7	211.4	217.2	195.0
		*r*_4_	157.1	194.6	184.4	180.5	172.3	188.0	190.9	187.4
Strategy 2	GBLUP/PBLUP	*r*_1_	11.1	23.0	12.4	31.9	9.1	13.7	13.8	8.7
(Approach 1)		*r*_2_	5.9	22.4	18.8	31.0	9.5	14.8	13.1	10.1
		*r*_3_	32.4	32.2	35.8	38.7	31.0	32.2	30.1	28.1
		*r*_4_	-5.6	9.4	1.4	1.4	0.0	7.3	5.1	3.9
	BRR/PBLUP	*r*_1_	16.7	10.4	25.3	42.0	12.9	17.7	13.8	14.0
		*r*_2_	8.0	9.0	27.3	33.3	38.1	9.1	9.5	6.8
		*r*_4_	-6.9	-0.8	1.4	0.0	0.0	5.8	3.7	3.9
	BL/PBLUP	*r*_1_	11.1	7.2	25.3	31.9	5.4	7.7	11.0	6.1
		*r*_2_	16.7	22.4	14.6	26.2	35.7	11.0	13.1	5.2
		*r*_4_	-8.3	5.0	1.4	0.0	-2.8	2.7	3.7	2.44
Strategy 2	GBLUP/PBLUP	*r*_1_	81.8	101.2	83.8	115.8	78.6	86.0	86.3	77.9
(Approach 2)		*r*_2_	73.2	100.3	94.4	114.3	79.2	87.8	85.0	80.2
		*r*_3_	116.6	116.4	122.2	126.9	114.4	116.4	112.8	109.4
		*r*_4_	54.6	79.0	65.9	65.9	63.6	75.6	72.1	70.0
	BRR/PBLUP	*r*_1_	90.9	80.6	105.1	132.4	84.8	90.9	86.3	86.6
		*r*_2_	76.8	78.4	108.3	118.2	126.0	78.6	79.2	74.8
		*r*_4_	52.3	62.3	65.9	63.6	63.6	73.1	69.7	70.0
	BL/PBLUP	*r*_1_	81.8	75.4	105.1	115.8	72.4	76.2	81.6	73.6
		*r*_2_	90.9	100.3	87.4	106.5	122.1	81.7	85.0	72.1
		*r*_4_	50.0	71.8	65.9	63.6	59.1	68.0	69.7	67.6

## Discussion

After the genomic selection (GS) concept was proposed in 2001 [1], genomic prediction studies were initially implemented in dairy cattle. The execution of GS in animal and crop breeding programs, such as dairy cattle, oat, maize and wheat, increased genetic gains [44, 73]. Implementation of GS in tree breeding is underway with recent publications in eucalypts [61, 74–77], white spruce [78–80], black spruce (*Picea mariana* [Mill.] BSP) [60], interior spruce [39, 70], Norway spruce [68, 81, 82], loblolly pine [58, 83, 84], lodgepole pine (*Pinus contorta* Douglas) [85] and maritime pine [66, 67]. GS was adopted in tree breeding in the last decade and different methods to estimate prediction efficiencies or accuracies of the cross-validated genomic predictions models have been implemented [2, 86]. In the current study, prediction efficiencies were assessed based on the four most common methods used in tree breeding (i.e., *r*_1_,*r*_2_,*r*_3_ and *r*_4_). Results from previous studies showed, in most cases, only one or two of these methods, suggesting that there is not a single consensual method within tree breeding community to evaluate genomic prediction estimations (Table S1).

Predictive ability (*r*_1_) has been widely used but normally shows lower values than *r*_2_,*r*_3_ and *r*_4_ for different traits (Table S1) as it uses the individual phenotypes as approximation to TBV and could be comparable to heritability [62, 68, 78, 87, 88]. Predictive accuracy (*r*_2_) and theoretical accuracy (*r*_3_) have been used less frequently [40, 74, 78]; the former one is considered as an unbiased estimate of accuracy of selection from n-fold cross-validation, since the correlation between an individual phenotype and its TBV cannot be higher than the square root of heritability [52, 89]. The *r*_3_ [63] was usually used to evaluate the models with full datasets, however in the current study it was used in the cross-validation analyses as well, since different PEV were obtained for each fold, thus estimations can be evaluated in the same way as the remaining methods.

Another method extensively used has been the prediction accuracy (*r*_4_) which generally showed higher values than *r*_1_,*r*_2_,*r*_3_ (Table S1). Congruent with previous studies [60, 65, 71, 80], we observed that all genomic models showed the highest values for *r*_4_ (0.65–0.75), followed by *r*_3_ (0.59–0.68), *r*_2_ (0.49–0.70), and *r*_1_ (0.19–0.43) that had the lowest values as expected. Nevertheless, using *r*_4_ (i.e., *corr*(*EBV*_*VP*_,*PEBV*_*y*_)) may inflate the prediction efficiency due to that the individuals in the validation population used to estimate EBV (*EBV*_*VP*_) were a proportion of the individuals in the full dataset (*y*) used to estimate PEBV (*PEBV*_*y*_) and therefore the correlation between them was generally higher [52, 68, 88].

### Heritabilities

No clear pattern was detected between *r*_4_ and heritability estimates for maritime pine [67] and norway spruce [68]. Additionally, Grattapaglia and Resende [3] noticed that *r*_3_ did not significantly change under different simulated heritability scenarios. Whereas no trend was detected among *r*_4_ and trait heritabilities, a positive and strong linear trend between *r*_1_,*r*_2_,*r*_3_ and heritabilities was observed in the current study, i.e., traits with lower heritabilities (below 0.25) exhibited the lowest *r*_1_,*r*_2_ and *r*_3_ (Fig. 1). Higher prediction efficiencies were obtained for traits with moderate heritabilities (above 0.30) which is in line with the positive correlation between trait heritabilities and *r*_1_ reported in loblolly pine [58] and maritime pine [66]. Chen et al. [68] concluded that values of narrow-sense heritability were more similar to values of *r*_1_ than to *r*_4_, as *r*_1_ involves both phenotypic and genetic values, however using *r*_2_ instead of *r*_1_ could remove influence of heritability since it is considered an unbiased estimation of the accuracy of selection from n-fold cross-validation [52, 78, 89].

### Effect of the imputation method on the genomic prediction efficiencies

For species such as Scots pine with large and complex genomes [90] but without a reference genome, and with no SNP chips or exome panels developed, genotyping-by-sequencing (GBS) method is considered as an attractive alternative to perform GS studies. When using GBS data, large amounts of missing data are produced, thus filtering and imputation of SNP markers are critical steps [42]. In an interior spruce genomic prediction study with GBS data [39], it was observed that the imputation method used had influence in the quality of genomic predictions and concluded that EM and kNN-Fam imputation methods, provided the highest genomic prediction accuracies (*r*_4_). EM was the most efficient imputation method in a wheat breeding GS study [33] with GBS data. Our study partially supports those findings, since among the genomic prediction models used, slightly higher predictions were observed in terms of *r*_2_,*r*_3_, and *r*_4_, when EM imputation algorithm was combined with GBLUP and BRR. In contrast, *r*_1_ was almost equal for each trait when BL model was used regardless of the imputation method used. We speculate that the slighly better performance of GBLUP-EM and BRR-EM could be due to that GBLUP and BRR respectively assume that QTL allelic effects are normally and multivariate normally distributed, and in addition the EM imputation method uses a kinship-based imputation algorithm which also assumes that marker genotypes follow a multivariate normal distribution [22, 33]. Thus, the combination of the same assumptions during the imputation and breeding value estimations can result in higher prediction efficiencies.

### Effect of the model on the prediction efficiencies

Traits of interest in tree breeding programs have different genetic architecture; thus, different genomic prediction models to evaluate prediction efficiencies may be used [44]. In a two generations maritime pine genomic selection prediction study [66] was observed similar *r*_1_ among GBLUP, BRR and BL for growth and stem straightness traits, but with larger prediction bias (estimated by regressing the EBV on the GEBV in the validation set) when BL was used. In a different maritime pine study with three generations, larger prediction bias were detected for PBLUP than for GBLUP or BL [67]. Several statistical methods, namely, GBLUP, BRR, BL and RKHS, were compared in a Norway spruce study with relatively similar *r*_1_ (0.16–0.44) and *r*_4_ values (0.58–0.77) observed for all of them [68]. However in the same study PBLUP outperformed the genomic models in terms of *r*_4_. On the contrary, genomic models (GBLUP, rrBLUP, BL, and RKHS) performed better than PBLUP in terms *r*_1_ (0.27 and 0.12, respectively) in eucalyptus hybrids, yet pedigree errors were observed in the populations studied, resulting in the underestimation PBLUP estimates [61]. The authors contemplated the possibility that the marker data captured precisely the Mendelian sampling variation, therefore the genetic variation was based on the true proportion of the genome that was IBD or IBS among individuals.

Our study is in line with the studies mentioned above, since similar prediction efficiencies were observed regardless the genomic model used. PBLUP outperformed the genomic models in terms of *r*_4_ (0.75–0.84) for all traits, however in terms of *r*_1_,*r*_2_ and *r*_3_ GBLUP, BRR, BL and PBLUP showed similar prediction efficiencies for growth and wood quality traits (Table 2). In short, either GBLUP, BRR or BL provided similar prediction efficiencies for growth and wood quality traits in Scots pine.

### Effects of the training and validation populations sizes on prediction efficiencies

Previous studies stated that *r*_1_ and *r*_2_ increased as the size of the training set increased without reaching a plateau which differed from our findings. For instance, Tan et al. [61] detected that *r*_1_ ascended as the TP size rose for all models and traits evaluated in eucalypt hybrids. Similarly, Lenz et al. [60] asserted that *r*_4_ increased as the TP size augmented, however after assigning TP of 67% of individuals the increase of *r*_4_ was negligible. Nevertheless, some similarities were found with other studies, especially when utilizing GBLUP, for which *r*_4_ rose as the TP size increased, achieving similar *r*_4_ values for height when TP reached 80–90% of individuals, and 75–90% of individuals for wood quality traits [68]. In the current study, a TP size of 70–80% was enough to obtain similar values as the full TP size in terms of *r*_1_,*r*_2_,*r*_3_ and *r*_4_, depending on the trait (Fig. 2). In the studies cited above [60, 61, 68], it was observed that the number of trees per family had an effect on the GS efficiency; however, in the current study in which the number of trees per family was very low, it was still observed the advantage of applying GS prediction methods in Scots pine.

### Effect of the number of SNPs

In a general conifer breeding program simulation study Li et al. [57] detected an increase in the accuracy (correlation between GEBV and simulated TBV) of GEBV for traits with low and high heritability when the subset of SNP markers increased from 7K to 90K, for a TP with 1000 clones from five simulated generations. Moreover, the same pattern was observed for GBLUP, BRR, BL and RKHS models in Norway spruce [68], where *r*_1_ and *r*_4_ increased with number of markers reaching almost a plateau between 4K and 8K SNP markers, regardless of the model used. Similarly, in eucalypt hybrids [61], when the subset of SNP markers dropped below 5K larger reduction in the *r*_1_ was observed for GBLUP and RKHS models; further, traits with lower heritabilities were observed to be more sensitive to the reduction in the number of SNP markers. On the contrary, in black spruce when markers were reduced randomly from 5K to 1K no noticeable decrease was found in *r*_4_ for GBLUP and Bayesian framwork models [60]; nonetheless, when markers were further reduced to 500, the *r*_4_ decreased dramatically.

Our results were in accordance with those studies, reaching similar efficiencies in terms *r*_1_ and *r*_2_ when the number of SNPs reached 6K–7K, or 3K–7K for *r*_4_ (Fig. 3), to those achieved when using all 8719 SNPs and regardless of the genomic model used, therefore the number of SNPs had more influence on the prediction efficiency than the genomic model used.

### Relative selection response of GS

A simulation study showed that when the breeding cycle length was reduced by 50% the *RSR*_*GS*:*PS*_ doubled, and that when the cycle length was reduced by 75% the *RSR*_*GS*:*PS*_ tripled at high marker levels [3]. This theory was confirmed by Resende et al. [91] that by reducing 50% the loblolly pine breeding cycle, reported a percentage of increase in selection efficiency of GS between 53–92% for DBH and 58–112% for Ht, compared to the traditional pedigree-based selection. Similarly, percentages of increase in GS efficiency varied between 106% to 139% for Ht when the breeding cycle length of interior spruce was reduced by 25% [70]. In Norway spruce, the percentages of increase in GS efficiency of MOE were between 69–83% when the cycle length was also shortened by 50% [68]. The results of the current study exhibited that a reduction of the cycle length by 50% increased the percentage GS efficiency to double for almost all traits, regardless the selection strategy (Table 3). Such reduction in the breeding cycle length of Scots pine could only be possible by shortening field-testing periods aided by the use genomic prediction at young ages, and that female flowering can start at earlier ages after greenhouse flowering stimulation [72]. Moreover, if cycles could even be shortened more than 50%, higher percentages of increase in GS efficiency could be reached which in the case of this study were almost triple than traditional pedigree-based selection (Table 3).

## Conclusions

Our results provide an initial perspective in the use of genomic prediction in Scots pine and are encouraging to develop GS strategies for the species. Similar prediction efficiencies were observed among pedigree and all genomic prediction models for growth and wood quality traits, suggesting that genomic prediction methods can be applied as an alternative to traditional pedigree predictions for Scots pine.

Our study showed that GS could potentially reduce the breeding cycle by half, and under that assumption, the relative genomic selection efficiency could double depending on the selection strategy and the trait.

The results presented here are based on a relatively small population with a shallow pedigree, for which 8K SNPs were sufficient to reach high GS prediction efficiencies. More studies using different populations, preferably populations with deeper pedigrees should be carried out to better understand the predictive power of SNP markers for traits with complex inheritance patterns in the species. The predictive power of SNP markers should be tested over at least two generations because the marker-QTL phase is expected to change once the population undergoes through breeding, due to recombination of homologous chromosomes during the meiosis.

## Methods

### Plant material

In this study a Scots pine full-sib progeny trial (identified as F261-Grundtjärn), belonging to the Swedish tree improvement program at Skogforsk (The Forestry Research Institute of Sweden) was used. The trial consists of 184 full-sib families and 7240 trees (F1-generation), generated from a partial diallel mating design of 40 plus-trees (F0-generation). The progeny trial was established in 1971 by Skogforsk as a randomized single tree plot design, divided into 14 post-blocks [92]. A more detailed description of the trial can be found in Fries et al. [93]. A number of 694 progeny trees (F1) from 183 families were selected for this study, such that the number of trees per family varied from one to seven with an average of four individuals per family.

### Phenotypic data

Growth traits were measured in the 7240 progeny trees whereas wood properties were estimated in a subset of 694 progeny trees. Height (Ht) was measured when the progeny trees were 10 (Ht1) and 30 (Ht2) years old. Diameter at breast height (DBH) was also measured twice, at ages 30 (DBH1) and 36 (DBH2). In 2011, increment cores at breast height were extracted and processed by Silviscan (Innventia AB, Stockholm, Sweden). From the Silviscan analysis, three traits were used in this study: microfibril angle (MFA), static modulus of elasticity (MOEs) and wood mean density (DEN). In addition, dynamic modulus of elasticity (MOEd) predicted by Hitman ST300 (Fiber-gen, Christchurch, New Zealand) was also used in the current study. Wood traits are further described in Hong et al. [94].

### DNA extraction and genotyping

The commercial NucleoSpin ^*Ⓡ*^ Plant II kit (Machery-Nagel, Dren, Germany) was used to extract genomic DNA from vegetative buds or needles from the 694 progeny trees and 46 parents. DNA concentration was determined with Qubit ^*Ⓡ*^ 2.0 fluorometer (Invitrogen, Carlsbad, CA, USA). Then, three genomic libraries for GBS were prepared following the procedure described in Pan et al. [29] by using 827 samples (replicates included) and PstI high fidelity restriction enzyme (New England Biolabs, MA, USA). The libraries were sequenced on an Illumina HiSeq 2000 platform at SciLifeLab, Sweden.

Thereafter, paired-end raw reads of each GBS library were cleaned and demultiplexed by the *process_radtags* module of Stacks v.1.40 [95] on the basis of 300 barcodes with 48 bp. Cleaned reads of each sample were aligned to the *Pinus taeda* v1.0 [96] reference genome, using BWA mem v0.7.15 [97] with default parameters. Alignments were coordinate-sorted and indexed using Samtools v1.5 [98]. SNP markers were called using the *mpileup* command of SAMtools over all the samples simultaneously, with default parameters, and converted into VCF matrix using BCFtools v0.1.19 [99]. Furthermore, these variants were sorted to keep only high-quality SNPs. Using *vcfutils* in BCFtools with default parameters, the SNPs within 3bp around an indel or with mapping quality <20 were filtered out; using Vcftools v.0.1.12b [100], only SNPs with coverage ≥5x, genotype quality (GQ) ≥30, genotype calling rate >20% were retained. Using the custom Perl program (ReplicateErrfilter.pl), discordant genotypes of 66 replicated samples were detected and the SNP sites with ≥3 replicate errors were filtered out. After this step, 24,152 informative SNP markers were retained.

Finally missing genotypic data were imputed using two imputation methods to compare their prediction efficiencies. Random (RND) imputation with the *codeGeno* function in synbreed package [101] in R (R Core Team 2016) and imputation with the expectation maximization (EM) algorithm by the *A.mat* function implemented in rrBLUP package [32] in R. A total of 15,537 and 15,433 SNPs with minor allele frequency (MAF) lower than 1% and with a missing data threshold lower than 10% were removed using RND and EM imputation methods, respectively.

### Statistical analysis

**Initial analysis.** Growth traits (Ht1, Ht2, DBH1 and DBH2) were available for all progeny trees in the trial, therefore univariate single site spatial analysis were performed in ASReml 4 standalone [64], with the objective to reduce the within-trial micro-environmental effects prior to any other analysis (see supplementary information S1). Briefly, diagnostic tools, variograms and plots of spatial residuals were used to detect design, treatment, local and extraneous effects. The predicted design effects and spatial residuals were extracted from the ASReml output files and used to remove micro-environmental effects from the raw data [102, 103]. Wood properties were assessed for a subset of 694 progeny trees and the micro-environmental effects were scaled for the raw data by removing the variation of the experimental design features and post-block effects. The environmentally adjusted phenotypic data (predicted values of each tree) were used for the genetic analysis [104–107].

**Best Linear Unbiased Prediction (BLUP).** The following model was used for PBLUP and GBLUP:

1 $$\begin{array}{@{}rcl@{}} y=Xb+Za+e, \end{array} $$

where *y* is the vector of the adjusted phenotypic data for each trait, *b* is the vector of fixed effects (intercept), *a* is the vector of random effects and *e* is the vector of residual effects, which is assumed to follow a normal distribution as $var(e)\sim N\left (0, I \sigma _{e}^{2} \right)$, where $\sigma _{e}^{2}$ is the residual variance and *I* is the identity matrix. *X* and *Z* are the incident matrices of *b* and *a*.

In the PBLUP, the vector *a* (additive genetic effects) from Eq. 1 is assumed to follow a normal distribution with expectations of $\sim N\left (0, A \sigma _{a}^{2} \right)$, where $\sigma _{a}^{2}$ is the additive genetic variance and *A* is the numerator relationship matrix (NRM). Briefly, the diagonal elements (*i*) of *A* were estimated according to Lynch and Walsh [108] as:

2 $$\begin{array}{@{}rcl@{}} A_{ii} = 1 + \frac{A_{gh}}{2}, \end{array} $$

where *g* and *h* are the parent of individual *i*.

The off-diagonal elements are the relationship between individuals *i* and *j* and were estimated as:

3 $$\begin{array}{@{}rcl@{}} A_{ij} = A_{ji} = \frac{A_{jg} + A_{jh}}{2}, \end{array} $$

For the GBLUP, the vector *a* is assumed to follow a normal distribution with expectations of $\sim N\left (0, G \sigma _{a}^{2} \right)$,where *G* is the genomic realized relationship matrix (RRM) estimated according to VanRaden [49] as:

4 $$\begin{array}{@{}rcl@{}} G=\frac{(M-P) (M-P)^{T}}{2\sum_{j}p_{j}\left(1-p_{j}\right)}, \end{array} $$

where *M* is the matrix of genotyped samples, *P* is the matrix of allele frequencies with the jth column given by 2(*p*_*j*_−0.5), where *p*_*j*_ is the observed allele frequencies of the genotyped samples. The elements of *M* were coded as 0, 1 and 2 (i.e., the number of minor alleles) for the estimation of the *G* matrix with function *kin* from the synbreed package in R in the case of RND imputed data, and with the function *A.mat* from the rrBLUP package in R, for the EM imputed data. PBLUP and GBLUP analyses were conducted in ASReml-R version 4.1.0.106.

**Bayesian models.** BRR and BL were implemented using the BGLR function from the BGLR package in R [109]. In brief, the following model was used:

5 $$\begin{array}{@{}rcl@{}} y=1_{n}\mu+Wm+e, \end{array} $$

where *y* is the vector of *n* adjusted phenotypes, 1_*n*_ is the vector of ones, *μ* is a scalar denoting the intercept, *W* is the incidence matrix for the *m* vector of marker effects, and *e* is the vector of residual effects that follow a multivariate normal distribution $e\sim N\left (0,I_{n}\sigma _{e}^{2}\right)$. In BRR, vector *m* from Eq. 5 is assigned a multivariate normal prior distribution with a common variance to all marker effects, that is $m\sim N\left (0,I_{p}\sigma _{m}^{2}\right)$, where *p* is the number of markers, $\sigma _{m}^{2}$ is the unknown genetic variance which is contributed by each marker and assigned as $\sigma _{m}^{2}~\chi ^{-}2(df_{m},S_{m})$, where *df*_*m*_ is degrees of freedom and *S*_*m*_ is the scale parameter. Residual variance is assigned as $\sigma _{e}^{2}~\chi ^{-}2\left (df_{e},S_{e}\right)$, with *df*_*e*_ degrees of freedom and scale parameter for residual variance *S*_*e*_ [55]. For the BL method assumes that vector *m* from Eq. 3 follows a hierarchical prior distribution with $m\sim N\left (0,T\sigma _{m}^{2}\right)$, where $T=diag\left (\tau _{1}^{2},\ldots,\tau _{p}^{2} \right)$. $\tau _{j}^{2}$ is assigned as $\tau _{j}^{2}~Exp\left (\lambda ^{2}\right), j\,=\,1,\ldots,p$. *λ*^2^ is assigned as *λ*^2^∼*Gamma*(*r*,*δ*). Finally, the residual variance is assigned as $\sigma _{e}^{2}\sim \chi ^{-}2\left (df_{e},S_{e} \right)$, where *df*_*e*_ is degrees of freedom and *S*_*e*_ is the scale parameter for residual variance [54].

For the Bayesian methods, GEBV in the VP were estimated as,

6 $$\begin{array}{@{}rcl@{}} \widehat{g}_{i}=\sum_{j=1}^{n}Z_{ij}^{'}\widehat{a_{j}}, \end{array} $$

where $Z_{ij}^{'}$ is the indicator covariate (-1, 0, 1) for the *i*^*th*^ tree at the *j*^*th*^ locus and $\widehat {a_{j}}$ is the estimated effect at the *j*^*th*^ locus.

**Model convergence and prior sensitivity analysis.** Bayesian algorithms were extended using Gibbs sampling for estimation of variance components. The Gibbs sampler was run for 20,000 iterations with a burn-in of 1,000 iterations and a thinning interval of 100. The convergence of the posterior distribution was verified using trace plots.

### Validation and evaluation methods

**Cross validation.** For all traits, pedigree based (PBLUP), genomic models (GBLUP, BRR and BL) and imputation method (EM and RND), a 10-fold cross-validation analysis was implemented, i.e., 90% of individuals randomly selected for the TP and 10% in the VP. BRR and BL were tested with eleven different sets of SNP markers randomly selected (i.e., 100, 200, 500, 1K, 2K, 3K, 4K, 5K, 6K, 7K and 8719). Additionally, to evaluate the performance of the pedigree versus genomic prediction models, different sizes of TP and VP were used. All individuals were randomly split into four different proportions of TP/VP, 80%, 70%, 60% and 50% (i.e., 555, 486, 417 and 347 individuals, respectively) for TP and the rest as VP. Each analysis was replicated 10 times.

**Prediction efficiency of traditional and genomic genetic evaluations.** Prediction efficiencies of pedigree and genomic models were evaluated and compared based on the predictive ability, predictive accuracy, theoretical accuracy, and prediction accuracy.

1. The predictive ability (*r*_1_) was defined as the Pearson product-moment correlation between the EBV of the individuals in the VP (*EBV*_*VP*_) and their adjusted phenotypes (*y*). i.e., *r*_1_=*corr*(*EBV*_*VP*_,*y*).
2. The predictive accuracy (*r*_2_) was estimated as the *r*_1_ scaled by *h* (square root of individual narrow sense heritability), i.e., *r*_2_=*corr*(*EBV*_*VP*_,*y*)/*h* [52].
3. The theoretical accuracy (i.e., square root of reliability) was estimated for PBLUP and GBLUP as $r_{3}=\sqrt {1-\frac {PEV}{G_{ii} \sigma _{a}^{2}}}$, where *PEV* is the prediction error variance of the VP, and *G*_*ii*_ is the diagonal element of the ith individual in the G matrix for GBLUP model or in the case of PBLUP model *G*_*ii*_=*A*_*ii*_, i.e., the diagonal element of A matrix [63].
4. The prediction accuracy (*r*_4_) was estimated as the Pearson product-moment correlation between the EBV of the individuals in the VP (*EBV*_*VP*_) and the PEBV estimated with all 697 progeny trees (*PEBV*_*y*_), i.e., *r*_4_=*corr*(*EBV*_*VP*_,*PEBV*_*y*_) [62, 91].

To avoid fold effects, all the methods were estimated within each fold and averaged across folds and replicates [53].

**Heritability estimation.** Pedigree- and genomic-based narrow sense heritabilities (*h*^2^) were estimated for PBLUP and GBLUP as:

7 $$\begin{array}{@{}rcl@{}} \widehat{h^{2}}=\frac{\widehat{\sigma_{a}^{2}}}{\widehat{\sigma_{p}^{2}}} = \frac{\widehat{\sigma_{a}^{2}}}{\widehat{\sigma_{a}^{2}}+\widehat{\sigma_{e}^{2}}}, \end{array} $$

where $\widehat {\sigma _{a}^{2}}, \widehat {\sigma _{p}^{2}}$ and $\widehat {\sigma _{e}^{2}}$ respectively are the additive genetic, phenotypic and residual variances.

**Relative selection response of GS.** Assuming that selection response is inversely proportional to the length of the breeding cycle, the relative selection response (*RSR*) of GS to the traditional pedigree-based selection (PS) can be estimated as a ratio (*RSR*_*GS*:*PS*_) between the efficiency or accuracy method and the breeding cycle time in years [3].

8 $$\begin{array}{@{}rcl@{}} RSR_{GS:PS}=\frac{r_{GS}}{r_{PS}}\times\frac{CL_{PS}}{CL_{GS}}, \end{array} $$

where *r*_*GS*_ and *r*_*PS*_ are the efficiency of GS and PS, respectively and *CL*_*PS*_ and *CL*_*GS*_ are the breeding cycle lengths of PS and GS, respectively.

The percentage of increase in selection efficiency of GS was estimated as (*RSR*_*GS*:*PS*_−1)∗100 [3]. In order to estimate *RSR*_*GS*:*PS*_, two GS approaches were assumed to reduce the breeding cycle, by shortening the period of field progeny testing needed for phenotypic evaluations. In addition, for the first approach the cycle length was reduced to 18 years considering that female flowering starts at 15–18 years in Scots pine [24], whereas the second approach assumed that flowering greenhouse stimulation [72] would produce female flowering at around 11 years.

*RSR*_*GS*:*PS*_ was estimated considering the four different prediction efficiency methods described in the previous sections (*r*_1_,*r*_2_,*r*_3_ and *r*_4_) and both breeding cycle reduction approaches.

## Supplementary Information

**Additional file 1** Supplementary information. The additional file contains detailed information about the initially performed spatial analysis and the Table S1 (prediction efficiencies for different tree species).

## Data Availability

The data-sets used and/or analyzed during the current study are available from Skogforsk and the corresponding author on reasonable request for research purposes.

## Abbreviations

GS: Genomic selection
MAS: Marker assisted selection
QTL: Quantitative trait loci
LD: Linkage disequilibrium
TP: Training population
VP: Validation population
GEBV: Genomic estimated breeding values
NGS: Next generation sequencing
SNP: Single nucleotide polymorphism markers
GBS: Genotyping-by-sequencing
MI: Mean imputation
EM: Expectation maximization
kNN-Fam: Family-based k-nearest neighbor
SVD: Singular value decomposition
GWAS: Genome-wide association
NRM: Numerator relationship matrix
BLUP: Best linear unbiased prediction
EBV: Estimated breeding values
RRM: Realized relationship matrix
IBD: Identical-by-descent
GBLUP: Genomic BLUP
BL: Bayesian-LASSO
GRM: Numerator relationship matrix
BRR: Bayesian ridge regression
Ne: Population size
TBV: True breeding values
PEBV: Pedigree based EBV
PBLUP: Pedigree based BLUP
RKHS: Reproducing kernel hilbert space
rrBLUP: Ridge regression BLUP
IBS: Identical-by-state
Ht: Tree height
DBH: Diameter at breast height
MFA: Microfibril angle
MOEs: Static modulus of elasticity
MOEd: Dynamic modulus of elasticity
DEN: Density
RND: Random
MAF: Minor allele frequency.

## References

[1] Meuwissen T, Hayes B, Goddard M (2001). Prediction of total genetic value using genome wide dense marker maps. Genetics.

[2] Grattapaglia D, Silva-Junior OB, Resende RT, Cappa EP, Müller BSF, Tan B, Isik F, Ratcliffe B, El-Kassaby YA (2018). Quantitative genetics and genomics converge to accelerate forest tree breeding. Front Plant Sci.

[3] Grattapaglia D, Resende MDV (2011). Genomic selection in forest tree breeding. Tree Genet Genomes.

[4] Goddard ME, Hayes BJ (2007). Genomic selection. J Anim Breeding Genet.

[5] Isik F, Whetten R, Zapata-Valenzuela J, Ogut F, McKeand S (2011). Genomic selection in loblolly pine - from lab to field. BMC Proceedings.

[6] Dekkers JCM (2007). Prediction of response to marker-assisted and genomic selection using selection index theory. J Anim Breeding Genet.

[7] Hayes BJ, Bowman PJ, Chamberlain AC, Verbyla K, Goddard ME (2009). Accuracy of genomic breeding values in multi-breed dairy cattle populations. Genet Sel Evol.

[8] Lorenz AJ, Chao s., Asoro FG, Heffner EL, Hayashi T, Iwata H, Smith KP, Sorrells ME, Jannink J-L (2011). Genomic selection in plant breeding: Knowledge and prospects. Adv Agron.

[9] Deschamps S, Campbell MA (2010). Utilization of next-generation sequencing platforms in plant genomics and genetic variant discovery. Mol Breeding.

[10] Varshney RK, Nayak SN, May GD, Jackson SA (2009). Next-generation sequencing technologies and their implications for crop genetics and breeding. Trends Biotechnol.

[11] Pérez-Enciso M, Rincón JC, Legarra A (2015). Sequence-vs. chip-assisted genomic selection: accurate biological information is advised. Genet Sel Evol.

[12] Birol I, Raymond A, Shaun DJ, Pleasance S, Coope R, Taylor GA, Yuen MMS, Keeling CI, Brand D, Vandervalk BP (2013). Assembling the 20 gb white spruce (*Picea glauca*) genome from whole-genome shotgun sequencing data. Bioinformatics.

[13] Nystedt B, Street N, Wetterbom A, Zuccolo A, Lin Y-C, Scofield DG, Vezzi F, Delhomme N, Giacomello S, Alexeyenko A (2013). The norway spruce genome sequence and conifer genome evolution. Nature.

[14] Neale DB, Wegrzyn JL, Stevens KA, Zimin AV, Puiu D, Crepeau MW, Cardeno C, Koriabine M, Holtz-Morris AE, Liechty JD, et al. Decoding the massive genome of loblolly pine using haploid DNA and novel assembly strategies. Genome Biol. 2014;15(R59). 10.1186/gb-2014-15-3-r59. 10.1186/gb-2014-15-3-r59 PMC4053751 24647006

[15] Zimin A, Stevens KA, Crepeau MW, Holtz-Morris A, Koriabine M, Marçais G, Puiu D, Roberts M, Wegrzyn JL, de Jong PJ (2014). Sequencing and assembly of the 22-Gb loblolly pine genome. Genetics.

[16] Stevens KA, Wegrzyn JL, Zimin A, Puiu D, Crepeau M, Cardeno C, Paul R, Gonzalez-Ibeas D, Koriabine M, Holtz-Morris AE (2016). Sequence of the sugar pine megagenome. Genetics.

[17] Suren H, Hodgins KA, Yeaman S, Nurkowski KA, Smets P, Rieseberg LH, Aitken SN, Holliday J (2016). Exome capture from the spruce and pine giga-genomes. Mol Ecol Resour.

[18] Vidalis A, Scofield DG, Neves LG, Bernhardsson C, García-Gil MR, Ingvarsson PK. Design and evaluation of a large sequence-capture probe set and associated SNPs for diploid and haploid samples of Norway spruce (Picea abies). bioRxiv 291716. 2018. 10.1101/291716.

[19] Neves L, Davis J, Barbazuk B, Kirst M. Targeted sequencing in the loblolly pine (*Pinus taeda*) megagenome by exome capture. BMC Proc. 2011;5(O48). 10.1186/1753-6561-5-S7-O48.

[20] Chen C, Mitchell SE, Elshire RJ, Buckler ES, El-Kassaby YA (2013). Mining conifer’ mega-genome using rapid and efficient multiplexed high-throughput genotyping-by-sequencing (GBS) SNP discovery platform. Tree Genet Genomes.

[21] Telfer E, Graham N, Macdonald L, Li Y, Klápště J, Resende Jr M, Neves LG, Dungey H, Wilcox P (2019). A high-density exome capture genotype-by-sequencing panel for forestry breeding in *Pinus radiata*. PLoS ONE.

[22] Poland JA, Rife TW (2012). Genotyping-by-sequencing for plant breeding and genetics. Plant Genome.

[23] Houston Durrant T, De Rigo D, Caudullo G (2016). Pinus sylvestris in Europe: distribution, habitat, usage and threats. European Atlas of Forest Tree Species.

[24] Matyás C, Ackzell L, Samuel CJA. EUFORGEN technical guidelines for genetic conservation and use for Scots pine (Pinus sylvestris). Bioversity Int. 2004.

[25] Krakau UK, Liesebach M, Aronen T, Pques LE (2013). Scots pine (*Pinus sylvestris* l.). Forest Tree Breeding in Europe.

[26] Fridman J, Nilsson P. Forest statistics of Swedish forests. 2015. https://www.slu.se/en/Collaborative-Centres-and-Projects/the-swedish-national-forest-inventory/forest-statistics/forest-statistics/, Accessed 7 April 2020.

[27] Nilsson O, Lundmark T. Slu receives major grants for forest research. 2019. https://www.slu.se/en/ew-news/2019/1/slu-receives-major-grants-for-forest-research/, Accessed 7 September 2019.

[28] He J, Zhao X, Laroche A, Lu Z-X, Liu H, Li Z (2014). Genotyping-by-sequencing (GBS), an ultimate marker-assisted selection (MAS) tool to accelerate plant breeding. Front Plant Sci.

[29] Pan J, Wang B, Pei Z, Zhao W, Gao J, Mao J, Wang X (2015). Optimization of the genotyping-by-sequencing strategy for population genomic analysis in conifers. Mol Ecol Resour.

[30] Troyanskaya O, Cantor M, Sherlock G, Brown P, Hastie T, Tibshirani R, Botstein D, Altman RB (2001). Missing value estimation methods for DNA microarrays. Bioinformatics.

[31] Dempster AP, Laird NM, Rubin B (1977). Maximum likelihood from incomplete data via the EM algorithm. J R Stat Soc Ser B Methodol.

[32] Endelman JB (2011). Ridge regression and other kernels for genomic selection with R package rrBLUP. Plant Genome.

[33] Poland J, Endelman J, Dawson J, Rutkoski J, Wu S, Manes Y, Dreisigacker S, Crossa J, Sánchez-Villeda H, Sorrells M (2012). Genomic selection in wheat breeding using genotyping-by-sequencing. Plant Genome.

[34] Hall D, Zhao W, Wennstrm U, Gull BA, Wang X-R (2020). Parentage and relatedness reconstruction in *Pinus sylvestris* using genotyping-by-sequencing. Heredity.

[35] Gorjanc G, Cleveland MA, Houston RD, Hickey JM (2015). Potential of genotyping-by-sequencing for genomic selection in livestock populations. Genet Sel Evol.

[36] Liu A, Lund M, Boichard D, Karaman E, Fritz S, Aamand GP, Nielsen US, Wang Y, Su G (2020). Improvement of genomic prediction by integrating additional single nucleotide polymorphisms selected from imputed whole genome sequencing data. Heredity.

[37] Crossa J, Beyene Y, Kassa S, Pérez P, Hickey JM, Chen C, de los Campos G, Burgueño J, Windhausen VS, Buckler E (2013). Genomic rediction in maize breeding populations with genotyping-by-sequencing. G3: Genes Genomes Genet.

[38] Jarquín D, Kocak K, Posadas L, Hyma K, Jedlicka J, Graef G, Lorenz A (2014). Genotyping by sequencing for genomic prediction in a soybean breeding population. BMC Genomics.

[39] El-Dien OG, Ratcliffe B, Klápště J, Chen C, Porth I, El-Kassaby YA (2015). Prediction accuracies for growth and wood attributes of interior spruce in space using genotyping-by-sequencing. BMC Genomics.

[40] Li Y, Klápště J, Telfer E, Wilcox P, Graham N, Macdonald L, Dungey HS (2019). Genomic selection for non-key traits in radiata pine when the documented pedigree is corrected using DNA marker information. BMC Genomics.

[41] Elshire RJ, Glaubitz JC, Sun Q, Poland JA, Kawamoto K, Buckler E, Mitchell S (2011). A robust, simple Genotyping-by-Sequencing (GBS) approach for high diversity species. PLoS ONE.

[42] Dodds KG, McEwan JC, Brauning R, Anderson RM, van Stijn TC, Kristjánsson T, Clarke S (2015). Construction of relatedness matrices using genotyping-by-sequencing data. BMC Genomics.

[43] Rosvall O. Review of the swedish tree breeding program. Skogforsk, Uppsala, Sweden. 2011.

[44] Crossa J, Pérez-Rodríguez P, Cuevas J, Montesinos-López O, Jarquín D, de los Campos G, Burgueño J, González-Camacho JM, Pérez-Elizalde S, Beyene Y (2017). Genomic selection in plant breeding: methods, models, and perspectives. Trends Plant Sci.

[45] Isik F (2014). Genomic selection in forest tree breeding: the concept and an outlook to the future. New Forests.

[46] Goddard ME, Hayes BJ, Meuwissen TH (2011). Using the genomic relationship matrix to predict the accuracy of genomic selection. J Anim Breeding Genet.

[47] White IMS, Hill WG (2020). Effect of heterogeneity in recombination rate on variation in realised relationship. Heredity.

[48] Henderson CR (1985). Best linear unbiased prediction of nonadditive genetic merits in noninbred populations. J Anim Sci.

[49] VanRaden PM (2008). Efficient methods to compute genomic predictions. J Dairy Sci.

[50] Ødegård J, Meuwissen TH (2014). Identity-by-descent genomic selection using selective and sparse genotyping. Genet Sel Evol.

[51] Ødegård J, Meuwissen TH (2015). Identity-by-descent genomic selection using selective and sparse genotyping for binary traits. Genet Sel Evol.

[52] Meuwissen T, Hayes B, Goddard M (2013). Accelerating improvement of livestock with genomic selection. Annu Rev Anim Biosci.

[53] Isik F, Holland J, Maltecca C (2017). Genetic Data Analysis for Plant and Animal Breeding.

[54] Park T, Casella G (2008). The bayesian lasso. J Am Stat Assoc.

[55] Pérez P, de los Campos G, Crossa J, Gianola D (2010). Genomic-enabled prediction based on molecular markers and pedigree using the Bayesian linear regression package in R. Plant Genome.

[56] de los Campos G, Perez P, Vazquez A, Crossa J, van der Werf J, B H, Gondro C (2013). Genome-enabled prediction using the BLR (Bayesian Linear Regression) R-package. Genome-Wide Association Studies and Genomic Prediction. Methods in Molecular Biology (Methods and Protocols).

[57] Li Y, Dungey HS (2018). Expected benefit of genomic selection over forward selection in conifer breeding and deployment. PLoS ONE.

[58] Resende MFR, Muñoz P, Resende MDV, Garrick DJ, Fernando RL, Davis JM, Jokela EJ, Martin TA, Peter GF, Kirst M (2012). Accuracy of genomic selection methods in a standard data set of loblolly pine (*Pinus taeda* L,). Genetics.

[59] Goddard M (2009). Genomic selection: prediction of accuracy and maximisation of long term response. Genetica.

[60] Lenz PR, Beaulieu J, Mansfield SD, Clément S, Desponts M, Bousquet J (2017). Factors affecting the accuracy of genomic selection for growth and wood quality traits in an advanced-breeding population of black spruce (*Picea mariana*). BMC Genomics.

[61] Tan B, Grattapaglia D, Martins GS, Ferreira KZ, Sundberg B, Ingvarsson P (2017). Evaluating the accuracy of genomic prediction of growth and wood traits in two *Eucalyptus* species and their F1 hybrids. BMC Plant Biol.

[62] Zapata-Valenzuela J, Isik F, Maltecca C, Wegrzyn J, Neale D, McKeand S, Whetten R (2012). SNP markers trace familial linkages in a cloned population of Pinus taeda–prospects for genomic selection. Tree Genet Genomes.

[63] Mrode RA (2014). Linear Models for the Prediction of Animal Breeding Values.

[64] Gilmour AR, Gogel BJ, Cullis BR, Welham SJ, Thompson R. ASReml user guide release 4.1 structural specification. Hemel hempstead: VSN international ltd. 2015.

[65] Bouvet J, Makouanzi G, Cros D, Vigneron PH (2016). Modeling additive and non-additive effects in a hybrid population using genome-wide genotyping: prediction accuracy implications. Heredity.

[66] Isik F, Bartholomé J, Farjat A, Chancerel E, Raffin A, Sanchez L, Plomion C, Bouffier L (2016). Genomic selection in maritime pine. Plant Sci.

[67] Bartholomé J, Van Heerwaarden J, Isik F, Boury C, Vidal M, Plomion C, Bouffier L (2016). Performance of genomic prediction within and across generations in maritime pine. BMC Genomics.

[68] Chen Z, Baison J, Pan J, Karlsson B, Andersson B, Westin J, García-Gil MR, Wu HX (2018). Accuracy of genomic selection for growth and wood quality traits in two control-pollinated progeny trials using exome capture as the genotyping platform in Norway spruce. BMC Genomics.

[69] Klápště J, Suontama M, Dungey H, Telfer E, Graham N, Low C, Stovold G (2018). Effect of hidden relatedness on single-step genetic evaluation in an advanced open-pollinated breeding program. J Hered.

[70] Ratcliffe B, El-Dien O, Klápště J, Porth I, Chen C, Jaquish B, El-Kassaby YA (2015). A comparison of genomic selection models across time in interior spruce (*Picea engelmannii* × *glauca*) using unordered SNP imputation methods. Heredity.

[71] Thistlethwaite FR, Ratcliffe B, Klápště J, Porth I, Chen C, Stoehr M, El-Kassaby Y (2017). Genomic prediction accuracies in space and time for height and wood density of Douglas-fir using exome capture as the genotyping platform. BMC Genomics.

[72] Almqvist C (2018). Improving floral initiation in potted *Picea abies* by supplemental light treatment. Silva Fenn.

[73] Meuwissen T, Hayes B, Goddard M (2016). Genomic selection: A paradigm shift in animal breeding. Anim Front.

[74] Cappa EP, El-Kassaby YA, Muoz F, Garcia M, Villalba P, Klápště J, Poltri S (2018). Genomic-based multiple-trait evaluation in *Eucalyptus grandis* using dominant DArT markers. Plant Sci.

[75] Suontama M, Klápště J, Telfer E, Graham N, Stovold T, Low C, McKinley R, Dungey H (2018). Efficiency of genomic prediction across two *Eucalyptus nitens* seed orchards with different selection histories. Heredity.

[76] Ballesta P, Maldonado C, Pérez-Rodríguez P, Mora F (2019). SNP and haplotype-based genomic selection of quantitative traits in Eucalyptus globulus. Plants.

[77] Ballesta P, Bush D, Silva FF, Mora F (2020). Genomic predictions using low-density SNP markers, pedigree and GWAS information: a case study with the non-model species *Eucalyptus cladocalyx*. Plants.

[78] Lenz PRN, Nadeau S, Azaiez A, Gérardi S, Deslauriers M, Perron M, Isabel N, Beaulieu J, Bousquet J (2020). Genomic prediction for hastening and improving efficiency of forward selection in conifer polycross mating designs: an example from white spruce. Heredity.

[79] Ratcliffe B, El-Dien OG, Cappa EP, Porth I, Klápště J, Chen C, El-Kassaby Y (2017). Single-step BLUP with varying genotyping effort in open-pollinated Picea glauca. G3: Genes Genomes Genet.

[80] Beaulieu J, Doerksen T, Clément S, MacKay J, Bousquet J (2014). Accuracy of genomic selection models in a large population of open-pollinated families in white spruce. Heredity.

[81] Lenz PRN, Nadeau S, Mottet MJ, Perron M, Isabel N, Beaulieu J, Bousquet J (2019). Multi-trait genomic selection for weevil resistance, growth, and wood quality in Norway spruce. Evol Appl.

[82] Zhou L, Chen Z, Olsson L, Grahn T, Karlsson B, Wu H, Lundqvist S-O, García-Gil MR (2020). Effect of number of annual rings and tree ages on genomic predictive ability for solid wood properties of norway spruce. BMC Genomics.

[83] Zapata-Valenzuela J, Whetten RW, Neale D, McKeand S, Isik F (2013). Genomic estimated breeding values using genomic relationship matrices in a cloned population of loblolly pine. G3: Genes Genomes Genet.

[84] Munoz P, Resende Jr M, Huber D, Quesada T, Resende MDV, Neale DB, Wegrzyn JL, Kirst M, Peter GF (2014). Genomic relationship matrix for correcting pedigree errors in breeding populations: impact on genetic parameters and genomic selection accuracy. Crop Sci.

[85] Ukrainetz NK, Mansfield SD (2020). Assessing the sensitivities of genomic selection for growth and wood quality traits in lodgepole pine using Bayesian models. Tree Genet Genomes.

[86] Daetwyler HD, Calus MPL, Pong-Wong R, de los Campos G, Hickey JM (2013). Genomic prediction in animals and plants: simulation of data, validation, reporting, and benchmarking. Genetics.

[87] Thistlethwaite FR, El-Dien O, Ratcliffe B, Klápště J, Porth I, Chen C, Stoehr M, Ingvarsson P, El-Kassaby Y (2020). Linkage disequilibrium vs. pedigree: genomic selection prediction accuracy in conifer species. PLoS ONE.

[88] Klápště J, Dungey HS, Graham NJ, Telfer EJ (2020). Effect of trait’s expression level on single-step genomic evaluation of resistance to Dothistroma needle blight. BMC Plant Biology.

[89] Legarra A, Robert-Grani C, Manfredi E, Elsen JM (2008). Performance of genomic selection in mice. Genetics.

[90] Neale D, Kremer A (2011). Forest tree genomics: growing resources and applications. Nat Rev Genet.

[91] Resende Jr MFR, Muoz P, Acosta JJ, Peter GF, Davis JM, Grattapaglia D, Resende MDV, Kirst M (2012). Accelerating the domestication of trees using genomic selection: accuracy of prediction models across ages and environments. New Phytol.

[92] Ericsson T (1997). Enhanced heritabilities and best linear unbiased predictors through appropriate blocking of progeny trials. Can J For Res.

[93] Fries A (2012). Genetic parameters, genetic gain and correlated responses in growth, fibre dimensions and wood density in a Scots pine breeding population. Ann For Sci.

[94] Hong Z, Fries A, Wu HX (2014). High negative genetic correlations between growth traits and wood properties suggest incorporating multiple traits selection including economic weights for the future Scots pine breeding programs. Ann For Sci.

[95] Catchen JM, Amores A, Hohenlohe P, Cresko W, Postlethwait JH (2011). Stacks: building and genotyping loci de novo from short-read sequences. G3: Genes Genomes Genet.

[96] Wegrzyn JL, Liechty JD, Stevens KA, Wu L-S, Loopstra CA, Vasquez-Gross HA, Dougherty WM, Lin BY, Zieve JJ, Martínez-García PJ (2014). Unique features of the loblolly pine (*Pinus taeda* l.) megagenome revealed through sequence annotation. Genetics.

[97] Li H, Durbin R (2010). Fast and accurate long-read alignment with Burrows-Wheeler transform. Bioinformatics.

[98] Li H, Handsaker B, Wysoker A, Fennell T, Ruan J, Homer N, Marth G, Abecasis G, Durbin R (2009). The sequence alignment/map format and SAMtools. Bioinformatics.

[99] Narasimhan V, Danecek P, Scally A, Xue Y, Tyler-Smith C, Durbin R (2016). BCFtools/RoH: a hidden Markov model approach for detecting autozygosity from next-generation sequencing data. Bioinformatics.

[100] Danecek P, Auton A, Abecasis G, Albers CA, Banks E, DePristo MA, Handsaker RE, Lunter G, Marth GT, Sherry ST (2011). The variant call format and VCFtools. Bioinformatics.

[101] Wimmer V, Albrecht T, Auinger HJ, Schn CC (2012). Synbreed: a framework for the analysis of genomic prediction data using R. Bioinformatics.

[102] Dutkowski GW, Silva JC, Gilmour AR, Lopez GA (2002). Spatial analysis methods for forest genetic trials. Can J For Res.

[103] Dutkowski GW, Silva JC, Gilmour AR, Wellendorf H, Aguiar A (2006). Spatial analysis enhances modelling of a wide variety of traits in forest genetic trials. Can J For Res.

[104] Dutkowski G, Ivkovik M, Gapare WJ, McRae TA (2016). Defining breeding and deployment regions for radiata pine in southern Australia. New Forests.

[105] Calleja-Rodriguez A, Andersson Gull B, Wu HX, Mullin TJ, Persson T (2019). Genotype-by-environment interactions and the dynamic relationship between tree vitality and height in northern *Pinus sylvestris*. Tree Genet Genomes.

[106] Calleja-Rodriguez A, Li Z, Hallingbäck HR, Sillanpää MJ, Wu HX, Abrahamsson S, García-Gil MR (2019). Analysis of phenotypic- and Estimated Breeding Values (EBV) to dissect the genetic architecture of complex traits in a Scots pine three-generation pedigree design. J Theor Biol.

[107] Chen Z, Karlsson B, Wu HX (2017). Patterns of additive genotype-by-environment interaction in tree height of Norway spruce in southern and central Sweden. Tree Genet Genomes.

[108] Lynch M, Walsh B (1998). Genetics and Analysis of Quantitative Traits.

[109] Perez P, de los Campos G (2014). Genome-wide regression and prediction with the BGLR statistical package. Genetics.

