# Supplementary material for: Evaluation of the efficiency of genomic versus pedigree predictions for growth and wood quality traits in Scots pine
Source: BMC Genomics. 2020 Nov 16;21:796. doi: 10.1186/s12864-020-07188-4 (PMC7667760; doi:10.1186/s12864-020-07188-4)
Supplement: Supplementary file 1 — Additional file 1 Supplementary information. The additional file contains detailed information about the initially performed spatial analysis and the Table S1 (prediction efficiencies for different tree species). [file 12864_2020_7188_MOESM1_ESM.docx]

Table S1. Different prediction efficiency methods used in the tree breeding literature. Predictive ability ($r_{1}$), predictive accuracy ($r_{2}$), theoretical accuracy ($r_{3}$) and prediction accuracy ($r_{4}$).

| Specie | Traits | h^2^ | Prediction efficiency method | | | | Reference |
| --- | --- | --- | --- | --- | --- | --- | --- |
|  |  |  | $r_{1}$ | $r_{2}$ | $r_{3}$ | $r_{4}$ |  |
| Eucalyptus sp. | Growth and wood quality | . | . | . | . | 0.64 - 0.74 | Resende et al. (2012a) |
| *Eucalyptus grandis* | Growth | 0.17 - 0.48 | . | . | 0.50 - 0.71 | . | Cappa et al. (2017) |
| *Eucalyptus grandis* | Growth and stem straightness | 0.17 - 0.34 |  |  | 0.77 - 0.80 |  | Cappa et al. (2018) |
| *Eucalyptus grandis* × *Eucalytus camaldulensis* | Growth and wood quality | 0.06 - 0.49 | 0.12 - 0.53 | . | . | . | de Moraes et al. (2018) |
| *Eucalyptus nitens* | Diameter, stem straightness and malformation | 0.05 - 0.28 | . | . | 0.52 - 0.63 | 0.34 - 0.66 | Klapste et al. (2018) |
| *Eucalyptus nitents* | Growth and wood quality | 0.03 - 0.50 | . | . | 0.29 - 0.79 | 0.02 - 0.46 | Suontama et al. (2018) |
| *Eucalyptus urophylla* × *Eucalyptus grandis* | Tree height | 0.15 - 0.25 | 0.20 - 0.29 | . | . | 0.55 - 0.80 | Bouvet et al. (2016) |
| *Eucalyptus urophylla* × *Eucalyptus grandis* | Growth, basic density, and pulp yield | 0.05 - 0.46 | 0.25 - 0.29 | . | . | . | Tan et al. (2017) |
| *Eucalyptus urophylla* × *Eucalyptus grandis* | Growth, basic density, and pulp yield | 0.03 - 0.47 | 0.18 - 0.50 | . | . | . | Tan et al. (2018) |
| *Eucalyptus polybractea* | Foliar terpene | . | 0.11 - 0.79 | . | . | . | Kainer et al. (2018) |
| *Picea abies* (L.) Karst | Growth and wood quality | 0.15 - 0.49 | 0.16 - 0.44 | . | . | 0.58 - 0.77 | Chen et al. (2018) |
| *Picea abies* (L.) Karst | Growth and wood quality | 0.10 - 0.43 | 0.07 - 0.46 | . | . | . | Chen et al. (2019) |
| *Picea abies* (L.) Karst | Growth, wood quality and weevil resistance | 0 - 0.20 | 0.10 - 0.46 | 0.69 - 0.97 | . | . | Lenz et al. (2019) |
| *Picea abies* (L.) Karst | Wood properties | 0.11 – 0.69 | 0.13 – 0.29 | 0.35 – 0.46 | . | . | Zhou et al. (2020) |
| *Picea glauca* (Moench) Voss | Growth and wood properties | 0.04 - 0.57 | 0.12 - 0.35 | . | . | 0.13 - 0.44 | Beaulieu et al. (2014a) |
| *Picea glauca* (Moench) Voss | Growth and wood quality | 0.32 - 0.57 | . | . | . | 0.29 - 0.79 | Beaulieu et al. (2014b) |
| *Picea glauca* (Moench) Voss | Tree height and wood density | 0.13 - 0.61 | . | . | . | 0.44 - 0.77 | El-Dien et al. (2016) |
| *Picea glauca* (Moench) Voss | Tree height and wood density | 0.18 - 0.59 | . | . | 0.51 - 0.66 | . | Ratcliffe et al. (2017) |
| *Picea glauca* (Moench) Voss | Growth and wood quality | 0.06 - 0.41 | 0.28 - 0.70 | 0.14 - 0.74 | 0.63 - 0.81 | . | Lenz et al. (2020) |
| *Picea glauca* x *Picea engelmannii* | Tree height | 0.25 - 0.61 | . | . | 0.74 - 0.76 | 0.31 - 0.55 | Ratcliffe et al. (2015) |
| *Picea glauca* x *Picea engelmannii* | Growth and wood quality | 0.07 - 0.60 | . | . | . | 0.25 - 0.77 | El-Dien et al. (2015) |
| *Picea glauca* x *Picea engelmannii* | Tree height and wood density | 0.25 - 0.39 |  | . | . | 0.001 - 0.70 | El-Dien et al. (2018) |
| *Picea mariana* | Growth and wood quality | 0.29 - 0.83 | 0.34 - 0.57 | . | . | 0.74 - 0.84 | Lenz et al. (2017) |
| *Pinus contorta* Douglas | Growth and wood quality | 0.25 - 0.47 | . | . | . | 0.25 - 0.87 | Ukrainetz and Mansfield (2019) |
| *Pinus pinaster* Ait. | Growth and stem sweep | . | 0.38 - 0.55 | . | . | . | Isik et al. (2016) |
| *Pinus pinaster* Ait. | Growth and stem straightness | 0.17 - 0.32 | . | . | . | 0.52 - 0.82 | Bartholome et al. (2016) |
| *Pinus radiata* D. Don | Stem straightness, branch-cluster frequency, external resin bleeding and internal checking | 0.18 - 0.28 | . | . | 0.55 - 0.80 | 0.47 - 0.70 | Li et al. (2019) |
| *Pinus taeda* L. | Growth, wood quality, development, and disease resistance | 0.07 - 0.45 | 0.17 - 0.51 | 0.37 - 0.77 | . | . | Resende et al. (2012b) |
| *Pinus taeda* L. | Growth | . | 0.37 - 0.52 | . | 0.70 - 0.71 | 0.69 - 0.74 | Zapata-Valenzuela et al. (2012) |
| *Pinus taeda* L. | Tree height | 0.15 - 0.35 | 0.77 - 0.89 | . | . | 0.64 - 0.86 | Munoz et al. (2014) |
| *Pseudotsuga menziesii* (Mirb.) Franco | Tree height and wood density | . | . | . | 0.61 - 0.76 | 0.78 - 0.91 | Thistlethwaite et al. (2017) |
| *Pseudotsuga menziesii* (Mirb.) Franco | Juvenile height | . | 0.21 - 0.43 | . | 0.61 - 0.68 | 0.10 - 0.99 | Thistlethwaite et al. (2019) |

Narrow sense heritability is represented by the acronym h^2^
